# What's on your mind? The role of bystander behaviors in victims' cognitions about the cause of the bullying and its solution

**DOI:** 10.1111/jora.70172

**Published:** 2026-03-30

**Authors:** Lydia Laninga‐Wijnen, Daniël Graf, Karyn Healy, Takuya Yanagida, Christina Salmivalli, Claire F. Garandeau

**Affiliations:** ^1^ INVEST/Psychology University of Turku Turku Finland; ^2^ Department of Psychology University of Queensland Brisbane Queensland Australia; ^3^ Department of Developmental and Educational Psychology University of Vienna Vienna Austria

**Keywords:** appraisals, attributions, bullying, defending, helplessness, self‐blame, victimization

## Abstract

It is commonly assumed that victims' maladaptive cognitions concerning the cause of their victimization (self‐blame) or its potential solution (e.g., helplessness) contribute to psychological problems. Nevertheless, there is limited empirical research on the conditions that lead to the emergence of such cognitions. The present study investigates whether bystanders' behaviors during bullying incidents (bystanders joining the bullying *or* defending the victim) influence victims' attributions of the cause (self‐blame) and perceived solutions (i.e., internal or external solution, or helplessness) to bullying, both concurrently and over time. A total of *n* = 755 victims (*M*_age_ = 12.75, SD = 1.77; 54.8% girls) from 379 classrooms in 49 schools were drawn from a larger sample of *n* = 6537 students participating in the SOLID project. Concurrent regression analyses indicated that victims whose bystanders joined the bullying (*n* = 345) experienced higher self‐blame and helplessness at T1 compared with victims whose bystanders did not join the bullying (*n* = 364). Victims whose bystanders defended them (*n* = 458) experienced lower self‐blame and helplessness, and a stronger belief in an internal or external solution to the bullying, compared with victims whose bystanders did not defend them (*n* = 286). Latent change score models indicated that over time, victims whose bystanders joined the bullying experienced less favorable change (i.e., stronger increase, weaker decrease) in self‐blame over time than victims whose bystanders did not join the bullying. Defended victims slightly differed from non‐defended victims in some cognitions about the solution (e.g., lower helplessness), but *only* if their victimization decreased between T1 and T2. Thus, bystander behaviors may shape victims' cognitions in response to bullying incidents. Anti‐bullying intervention should emphasize that bystanders should not join in the bullying; further research is needed to clarify when and how bystanders' defending is helpful.

School bullying remains a significant issue globally. In Finland, where the current study was conducted, nearly one in five children reports to experience at least one form of bullying multiple times each month (Organisation for Economic Co‐operation and Development [OECD], 2020). Being a victim of bullying can lead to psychological problems, such as increased internalizing symptoms (Christina et al., 2021). According to the transactional model of stress and coping (Lazarus & Folkman, 1984) and other theories of cognitive vulnerability (Abramson et al., 1978), *cognitions* can play a key role in explaining why being victimized contributes to psychological problems. These include cognitions about (1) the *cause* of the victimization (causal attributions; Weiner & Graham, 1999), and (2) potential *solutions* to the victimization (e.g., beliefs on whether the situation can be solved, and if so, whether the solution is internal or external to the victim; Noret et al., 2018). Indeed, students who are more frequently victimized often blame themselves for their plight (e.g., “I am being bullied because I am not a fun child”; Schacter et al., 2015), which worsens their psychological problems over time (Perren et al., 2013). Moreover, victims of bullying can experience helplessness about their situation (e.g., believe that there is no solution), which increases depressive symptoms (Hunter et al., 2010). Victims vary greatly from each other in these cognitions (Laninga‐Wijnen, Garandeau, et al., 2024), but the reasons for this variation remain unexplored. According to the transactional model of stress (Lazarus & Folkman, 1984), cognitions in response to stressful situations are shaped through ongoing transactions between an individual and their environment. Bullying is often a group process; therefore, *the reactions of bystanders* may be an important environmental factor shaping victims' cognitions about their situation. Bystanders can choose to join the bully (e.g., by assisting or laughing about the situation) or defend the victim (e.g., by standing up against the bully; Salmivalli et al., 2011). The current study examines how these bystander reactions relate to victims' cognitions about the *cause of* and the *solution to* their victimization, both concurrently and over time.

## Do bystander behaviors predict victims' cognitions about the *cause* of the bullying?

According to attribution theory (Weiner, 2000) applied to the victimization context (Graham & Juvonen, 1998), victims commonly ask themselves: why is this happening to me? Thus, they wonder about the *cause* of their situation. The answer to this question can unfold along various dimensions (i.e., whether the cause is internal or external to the victim, perceived as stable, and considered controllable). Most research has focused on the first dimension, demonstrating that victims who blame themselves for their victimization (internal causal attribution) experience worse psychological problems (Graham & Juvonen, 1998; Perren et al., 2013).

The behavioral reactions of bystanders may shape victims' self‐blaming attributions. First, if bystanders *join the bully*, this signals to the victim that others consider the harassment to be justified. Attributional theory suggests that when others attribute a problem to a lack of effort of the person experiencing it (internal, controllable), this leads to punishment and reprimand (Weiner & Graham, 1999). Having bystanders join the bully may be interpreted by victims as such ‘reprimand’ and therefore as evidence that the victim is to blame for the situation. Second, if bystanders support the bully, the victimization may be especially *humiliating* for the victim, because there are witnesses encouraging the bullying (the ‘spectator effect’; Menesini & Camodeca, 2008; Nishina et al., 2010), and increased feelings of humiliation were found to relate to higher self‐blame (Laninga‐Wijnen et al., 2024b). Third, when bystanders join in the bullying, it emphasizes that these bystanders, unlike the victim, are *not* targeted. These upward social comparisons (Visconti et al., 2013) may reinforce victims' feeling that they are singled out for harassment, leading them to think that there must be something wrong with them. Indeed, cross‐sectional studies have shown that victims whose bystanders joined in the bullying had more psychological problems than victims who reported *no witnesses* when bullied (Jones et al., 2015; Nishina, 2012). Yet, to our knowledge, whether bystanders' joining in the bullying relates to victims' *self‐blame* has never been tested.

Alternatively, some bystanders may defend victims, for instance by telling the bully to stop (Salmivalli et al., 2011). Because such behavior is *directed at bullies*, it may communicate to the victim that the bullies' actions are perceived as unwarranted, and that bystanders consider the bullies—rather than the victims—as the ones to be blamed for the situation (Laninga‐Wijnen, Garandeau, et al., 2024). This may prevent victims from engaging in self‐blame by helping them put their victimization experience in perspective: not all peers subject them to harassment, nor consider this harassment to be justified, and some even attempt to stop it (Graham & Juvonen, 1998). Only two studies have examined the role of being defended in victims' self‐blame. A cross‐sectional daily diary study (Laninga‐Wijnen et al., 2024b) on partially the same sample as the current study found that 7th‐ to 9th‐ graders reported lower self‐blame on days they were victimized *and* defended as compared with days they were victimized but *non‐*defended. Yet, it is unknown whether the effect of being defended on daily fluctuations in self‐blame generalize to within‐person changes in self‐blame across longer time‐spans. Notably, a longitudinal study on 4th to 9th grade students (Laninga‐Wijnen, Garandeau, et al., 2024) found that in a sample of stable victims, being more frequently victimized related to *higher self‐blame* in classrooms with higher levels of defending. Although it is unclear whether these stable victims were the ones being defended in these classrooms, it is possible that victims increase in self‐blame if their victimization continues despite high classroom defending. They may start to wonder: “why am I still being victimized in this classroom where everybody stands up for each other? It must be something about me”. This latter study demonstrates the need to examine whether *victimization stability* moderates the prospective link between bystander behaviors and victims' self‐blame.

Our study builds upon this previous work by examining whether bystander behaviors (joining the bullying and defending the victim) predict victims' self‐blame, both concurrently and longitudinally (across a three‐month timespan). We anticipated that being defended relates to reduced self‐blame because it signals social support and may contribute to perceiving the cause of victimization as external. Yet, regarding the longitudinal link, we hypothesized a moderating effect of *changes* in victimization (cf. Laninga‐Wijnen, Garandeau, et al., 2024): defended victims may be more likely than non‐defended victims to decrease in self‐blame, but *only* if their victimization decreases. Instead, if the victimization of defended victims remains stable or even increases, they may think that there is something wrong with them (as indicated by an increase in self‐blame) which explains why they remain victimized despite others' efforts to end it. Furthermore, we examined whether bystanders joining the bullying increased victims' self‐blame over time, particularly if they remain victimized. That is, when the bullying continues, victims might believe that bystanders were justified in joining the bullying and increase their self‐blame. In contrast, when the bullying decreases, victims may question the bully‐supporting behaviors, potentially decreasing self‐blame.

## Do bystander behaviors predict victims' cognitions about the *solution* to the bullying?

Bystander behaviors may also affect victims' cognitions about the potential *solution* to their situation*—*that is, whether victims believe that they will be able to cope with their situation (either themselves or with the help of others) or instead, think that nothing can be done to stop the bullying (helplessness; Noret et al., 2018). The Transactional Model of Stress and Coping (Lazarus & Folkman, 1984) posits that the impact of stressful experiences is determined through ongoing transactions between individuals and their social environment, and that individuals' primary and secondary appraisals shape their emotional response to stressors. *Primary appraisals* include individuals' cognitions about how harmful, threatening or challenging the stressful situation is (Oliver & Brough, 2002), and *secondary appraisals* refer to individuals' cognitions regarding whether any actions can be taken to improve the situation and if so, which coping options would be most successful. Receiving social support during a stressful event can potentially affect both types of appraisals by (a) decreasing perceived harm or threat, and (b) informing about coping resources.

Peer victimization is a social stressor, posing a threat to individuals' self‐esteem and wellbeing (van Geel et al., 2018). Bystander reactions, that is, joining the bully or defending the victim, may not only predict the extent to which victimization is considered harmful or threatening (primary appraisal) but also inform victimized youth on available coping options to solve the bullying (secondary appraisal). The current study focuses on how bystanders affect these *secondary appraisals*, including (1) the victims' belief that the bullying could stop if others would help them (external solution; or “social efficacy”; Noret et al., 2018), (2) the victims' belief that they could stop the bullying themselves (internal solution—also referred to as “coping efficacy”, Bandura, 1997; or a lack of “personal helplessness”; Abramson et al., 1978), and (3) the victims' belief that *nothing* could stop the bullying (helplessness; also referred to as “universal helplessness”; Abramson et al., 1978).

When bystanders *join in the bullying*, victims may perceive the situation as more threatening and less controllable (primary appraisals), which informs victims' cognitions about the potential solution of their situation (secondary appraisal). They may believe that they are not capable of solving the situation themselves (no *internal solution*; Nishina, 2012) or even that there are no solutions to it at all (*helplessness*). Regarding the effect of bystanders joining in the bullying on victims' cognitions about *external solutions* (e.g., the bullying will stop with the help of others), opposing hypotheses can be made. On the one hand, the heightened power imbalance may lead victims to think that they need others, so as to outnumber the bullies and their allies (Xie et al., 2022), thus reinforcing their belief in an external solution. On the other hand, when bystanders join in the bullying, victims may increasingly distrust their peers and perceive them as *unwilling* to solve their situation. They may generalize these thoughts to any bystander. Moreover, being victimized by multiple perpetrators can lead to feelings of shame, which could deter victims from seeking help, suggesting they may lack confidence in an external solution (Cohen & Wills, 1985; Kim & Craig, 2024). To date, no study has examined whether having bystanders join the bullying predicts victims' cognitions about the solution to their situation.

Bystanders' *defending behaviors* may also predict victims' cognitions about the solution to their situation. First, defended victims may experience lower *helplessness*, because being defended may increase the number of pathways through which the bullying situation could potentially be solved (Ricker et al., 2022), highlighting available coping resources (Gazelle & Druhen, 2009; Lazarus & Folkman, 1984). Furthermore, defended victims may have stronger trust in *external solutions* (e.g., others will help me end my suffering), because they actually see bystanders trying to solve the situation.

Furthermore, opposing hypotheses can be made about how being defended affects victims' confidence in their own capacity to stop the bullying (i.e., *internal solution*). On the one hand, victims who have bystanders standing up for them may feel empowered and see these bystanders as role models from whom they can learn how to address the situation themselves (vicarious learning; self‐efficacy theory, Bandura, 1997). Consequently, defended victims may be more convinced than non‐defended victims that they will be able to stand up for themselves. On the other hand, it has been recently theorized that peer defense may increase victims' passivity in finding a solution to their situation themselves (Healy, 2020). Peers' defending behaviors may signal to victims that their peers believe that the victims are incapable of solving their situation themselves, making victims think that they have insufficient internal resources to handle their situation. Although this alternative hypothesis has not been examined in the area of school bullying, research in other fields suggests that help can sometimes undermine one's self‐efficacy in solving a problem. For instance, studies on aged care found that well‐intentioned assistance by staff can undermine independence and perceived self‐efficacy in solving problems (Foy & Mitchell, 1991). In workplace research, help that was not needed, or was offered before being requested, was interpreted by recipients as negative feedback about competence or status (Beehr et al., 2010; Harari et al., 2022). Similarly, an experimental study found that children who received help from the teacher were perceived as less competent by their classmates than other children (Sierksma & Shutts, 2020). Research into “miscarried help” reports that some parenting behaviors, intended to help sick children, may lead to poorer child adjustment over time by making the child unnecessarily dependent (Fales et al., 2014). Thus, an alternative possibility is that, as a result of bystander intervention, victims are *less* convinced that they will be able to address the bullying themselves (lower belief in an *internal solution*).

Longitudinally, it is likely that the effect of being defended on cognitions about the solution depends on whether the victimization decreases or not. Defended victims whose victimization remains stable or increases, should have higher feelings of helplessness and less strongly believe in an external solution, as compared with defended victims whose victimization decreases or ceases. We will explore the longitudinal role of being defended in victims' belief in internal solutions and the moderating role of victimization stability in this regard.

## Present study

**FIGURE 1:**
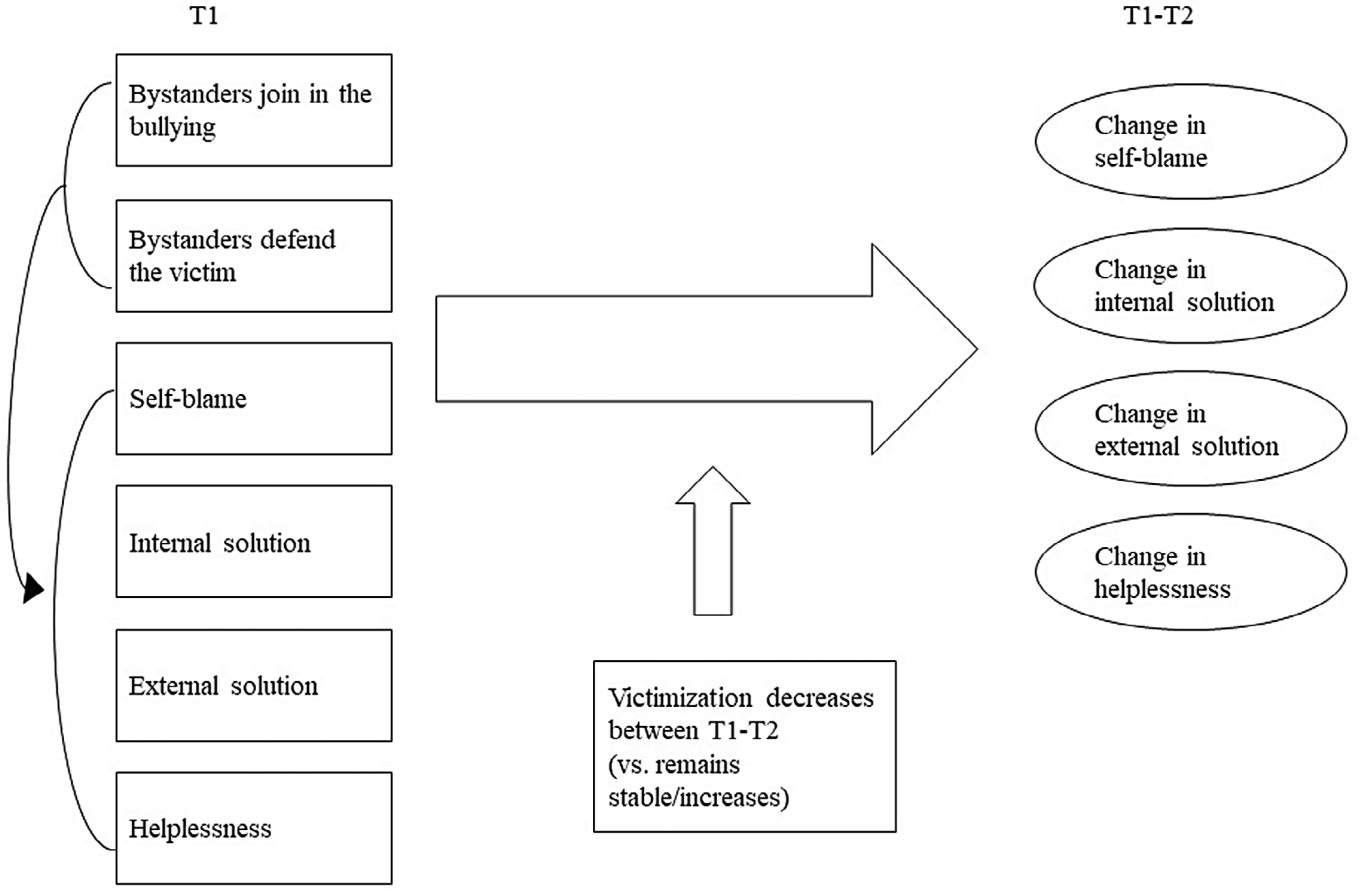
The role of bystander behaviors in victims' cognitions, concurrently (listed under T1) and over time (big arrow).

In a sample of victimized students, we will examine whether bystanders' responses to bullying predict victims' cognitions on the *cause* of their situation (self‐blame), and on their belief that there is an (internal or external) *solution* to the bullying or not (helplessness). We examine this both concurrently at the start of the school year (T1) *and* over time, from the start of the school year (T1) until halfway through the school year (T2). We focus on two types of bystander reactions: (1) joining the bullying and (2) defending the victim (see Figure 1).

### Cognitions about the cause

Concurrently, we expect that victims whose bystanders join in the bullying experience higher self‐blame than victims whose bystanders do not join in. Longitudinally, we expect victims whose bystanders join in the bullying to increase more strongly in self‐blame than victims whose bystanders do *not* join in the bullying. We expect this effect do be especially strong if victimization remains stable or increases.

Regarding defending, we expect defended victims to have lower self‐blame than non‐defended victims. Longitudinally, we expect that the effect of being defended depends on whether the victimization decreases or not: defended victims whose victimization decreases may be less likely to engage in self‐blame over time than defended victims whose victimization remains stable or increases.

### Cognitions about the solution

Concurrently, we expect victims whose bystanders join the bullying to have higher levels of *helplessness* and to be less likely to believe in an *internal solution* to their situation, compared with victims whose bystanders do *not* join the bullying. We explore the role of having bystanders join in the bullying on victims' belief in an *external solution* to their problem. Longitudinally, we expect that having bystanders join in the bullying decreases victims' belief in an internal or an external solution, and increase helplessness. We expect effects to be exacerbated for victims whose victimization remains stable or increases.

Regarding defending, we expect that, concurrently, defended victims report lower helplessness and a stronger belief in an external solution than non‐defended victims. Furthermore, we explore whether defended victims differ from non‐defended victims in their belief in an internal solution. Longitudinally, we hypothesize that the role of being defended in victims' cognitions about the solution depends on whether the victimization decreases or not. We hypothesize that defended victims whose victimization declines are more likely to decrease in helplessness and increase in their belief in an external solution to the bullying; in contrast, defended victims whose victimization remains stable or increased may experience increased helplessness and decrease in their belief in an internal or external solution to the bullying.

### Covariates: gender, age, victimization frequency, and depressive symptoms

In this study, we controlled for students' age, as research suggests that older students use more active coping strategies whereas younger students use more passive strategies when handling a stressful situation (Williams & McGillicuddy‐De Lisi, 1999). Thus, older students may be more likely to think that, to solve their situation, they need to actively seek support (external solution) or stand up for themselves (internal solution; Lazarus & Folkman, 1984). Second, we controlled for gender, as some work found girls to be more likely to engage in self‐blaming attributions than boys (Di Tata et al., 2025, but see Perren et al., 2013 who did not detect gender differences), and ask support from friends (Visconti et al., 2013). Third, we controlled for pre‐existing victimization at the first timepoint, because more frequently victimized youth were found to blame themselves more for their victimization in previous work (Laninga‐Wijnen, Garandeau, et al., 2024). More frequently victimized youth may also consider their situation as more challenging and threatening (primary appraisals), which typically limits their perceived coping options and thus increases helplessness and lack of belief in an internal *and* external solution (Singh & Bussey, 2011). Lastly, we controlled for students' depression at the first time point, because this typically relates to cognitive distortions, such as increased self‐blame (Schacter & Juvonen, 2017) and helplessness (Nixon et al., 2023).

## METHOD

### Participants

We used two waves of data (T1 = September/October, T2 = January) from *n =* 755 students who reported at T1 that they were victimized; This sample of victimized students was drawn from a large sample of *n* = 6357 Finnish 4th to 9th grade students participating in the SOLID project. More information on *how* these victims were selected from the general sample is provided in the procedure section. The 755 victims belonged to 379 classrooms in 49 schools. Participants were on average *M* = 12.75 years of age (*SD* = 1.77), and 54.8% identified as girl, whereas 0.8% identified as neither boy nor girl. There were on average *M* = 1.99 (range = 1–6) victims per class and *M* = 15.41 (range = 2–41) per school. All participating schools were public, reflecting the situation in Finland where the vast majority (>95%) of basic education schools are publicly funded. Each school included students from diverse socioeconomic backgrounds, with no substantial differences in this diversity across schools. Students typically attend their neighborhood school, and although some socioeconomic variation exists between neighborhoods, overall levels of segregation in Finland remain low. The Finnish welfare system aims to provide equal access to high‐quality education for all children, regardless of their place of residence.

### Procedure

Only students who received active consent from parents and also provided assent themselves participated in our study (56%). Data collection took place across two school years (2022–2023, Cohort 1; 2023–2024, Cohort 2). Online questionnaires were administered during regular teaching hours under the supervision of teachers who received thorough instructions two weeks before the data collection. Participants were assured of the confidentiality of their responses, informed about the voluntary nature of their participation, and told that they could withdraw at any point. The order of the administered scales was non‐randomized, so that defending items could be prompted *after* students responded to victimization items. The Ethical Board of the University of Turku granted approval for this project (nr. 53/2021).

In total, *n* = 6357 students belonging to 660 classrooms from 51 schools participated in the first two waves of the project. Based on their responses to the questionnaires about victimization and defending (see further description below), we selected students as *victims* if they: (a) experienced one form of victimization two or more times a month, or (b) experienced two or more forms of victimization at least once or twice in the past few months. However, victims were excluded from this sample if they consistently reported on follow‐up items, about bystander behaviors, that nobody had been mean to them nor bullied them (cf. Laninga‐Wijnen et al., 2026). In Supporting Information (Appendix S1), we provide more information regarding this selection process.

### Measures

#### Victimization frequency at T1 and T2

Students were introduced to a written definition of bullying that matched Olweus' (1978) definition (see Laninga‐Wijnen et al., 2025). Then, students were prompted with the general question “Have you been bullied at school like this during the last couple of months?”, followed by five specific victimization items, tapping into physical, verbal, relational, and material forms of victimization. Sample items are “I was called nasty names or laughed in my face” and “I was hit, kicked, or pushed”. Youth responded to the items on a 5‐point Likert scale, with 0 = *not at all*, 1 = *once or twice*, 2 = *two or three times a month*, 3 = *about once a week*, and 4 = *several times a week* (Olweus, 1978; Sjögren et al., 2025). Confirmatory factor analyses for T1 and T2 on the five items using the full sample participating in the project had reasonable fit (CFI = .990, TLI = .974, RMSEA = .054, SRMR = .015 for T1, and CFI = .994, TLI = .984, RMSEA = .047, SRMR = .011 for T2). The five‐item scale showed acceptable reliability, with *ω* = .77 at T1 and *ω* = .79 at T2. Moreover, we conducted longitudinal measurement invariance tests correcting for the clustering of individuals in classrooms to investigate configural (i.e., equality of factor structure), metric (i.e., equality of factor loadings), and scalar measurement invariance (i.e., equality of intercepts) across the two waves. Scalar measurement invariance was tenable with acceptable model fit (CFI = .982, TLI = .972, RMSEA = .035, SRMR = .031, ΔBIC = 23.97 for metric vs. scalar). Scale scores for T1 and T2 were generated by averaging the answers across the items, with higher scores representing higher victimization frequency.

#### Changes in victimization frequency between T1 and T2

Changes in victimization between T1 and T2 were included as a moderator in longitudinal analyses. To this end, difference scores were calculated by subtracting T2 values from T1 values. Negative values on these difference scores indicate an increase in victimization frequency between T1 and T2; scores of zero reflect stability, and positive scores suggest a decrease in victimization frequency. Thus, a higher score on this scale represents more *favorable changes in victimization*. Difference scores varied from −3.10 to 4.00 with an average of *M* = 0.38. To test our hypotheses about the dichotomous distinction between (1) students decreasing in victimization versus (2) students remaining stable/increasing in victimization, we decided to dichotomize the difference score. Students who scored ≥0.20 were categorized as ‘decreasing’ (coded as 1; 61.3% of the sample), because this represents the smallest decrease possible (implying that students score 1 point lower on one out of the five victimization items); whereas students scoring <.20 were categorized as ‘remaining stable/increasing’ (coded as 0; 38.7%). In sensitivity analyses, we used the continuous difference score to check the robustness of our findings.

#### Bystander behaviors at T1

Students' perceptions of bystander behaviors, when exposed to mean or bullying behaviors, were assessed using a novel scale found to be valid and reliable in previous work with a comparable sample (Laninga‐Wijnen et al., 2025). All items started with the sentence: “The past few months, when a fellow student was mean to me or bullied me”… followed by specific descriptions of how bystanders joined in the bullying or mean behaviors (2 items) or defended the participant (5 items). An example item for joining the bully was “…one or more of my classmates started being mean to me or bullying me, too”, and an example item for defending the victim was “…one or more of my classmates told the bully to stop”. For each item, students could answer with “*yes*” (1), “*no*” (0), or “not applicable because nobody was mean to me or bullied me” (coded as missing in this study). Confirmatory factor analyses showed that a two‐factor structure (one for joining in the bullying, one for defending the victim) yielded good fit in our sample (CFI = .988, TLI = .981, RMSEA = .042, SRMR = .061). We created a dummy variable “bystanders join bullying”, for which students received a ‘1’ if they had at least one classmate joining the bullying (48.7%), and a ‘0’ if no classmate joined the bullying across items (51.3%). For the dummy variable “bystanders defend victim”, students received a ‘1’ if they reported being defended on at least one item (61.6%), and a ‘0’ if they did not report being defended on any item (38.4%). About 30% of the students had both bystanders defending them *and* bystanders joining the bullying.

#### Cognitions about the cause of victimization at T1 and T2

The scale used to assess self‐blame was adapted from the “Why Kids Pick on Me” measure developed by Visconti et al. (2013). Participants were asked about various reasons why someone might pick on them. Four items assessed the extent to which students blamed themselves, including “because I am different from the bullies” and “because I do not wear cool clothes”. Items were answered on a 4‐point Likert scale (0 = *never the reason*, 3 = *always the reason*). Confirmatory factor analyses indicated that the four items loaded on one factor with good model fit at both T1 (CFI = .982, TLI = 1.000, RMSEA = .000, SRMR = .017) and T2 (CFI = .968, TLI = 1.000, RMSEA = .000, SRMR = .022), with factor loadings varying from .487 to .692 across waves. Longitudinal measurement invariance indicated that scalar fit was tenable, with CFI = .948, TLI = .890, RMSEA = .086, and SRMR = .047, and Δ BIC = −17.21 for scalar vs. metric. Reliabilities were *ω* = .78 at T1 and *ω* = .80 at T2. We averaged the items to create indices of self‐blame at T1 and T2.

#### Cognitions about the solution of victimization at T1 and T2

Six newly developed items were administered to assess students' cognitions about the solution to their situation. These items were partially based on the Self‐Reliance subscale of the BASC‐2 and tailored to the context of bullying (see also Parris et al., 2019). The questionnaire about these cognitions started with the sentence “If somebody would bully me…”, followed by various items on cognitions about internal solutions and external solutions, as well as some reverse‐coded items. Students could respond on a scale from 0 = *not true at all* to 3 = *totally true*.

Because this was a new scale, we conducted exploratory maximum likelihood factor analyses, to determine the number of factors that could be distinguished based on these items. Both at T1 and at T2, a three‐factor solution was found to be superior over a two‐factor solution Δ*χ* ^2^(4) = 48.99, *p* < .001. The model fit for the three‐factor solution was good, with RMSEA = .000 and SRMR = .001 at W1, and RMSEA = .000 and SRMR = .004 at T2. The first factor represented a disbelief that there would be a solution to the bullying (“helplessness”), and included the items “I would feel helpless” and “I would not be able to stop the bullying, whatever I do”. The second factor represented students' confidence in their own ability to stop the bullying themselves (“internal solution”), including the items “I know how I can react to the bully so that the bullying will stop” and “I would stand up for myself against the bully”. The third factor represented students' confidence in others' ability to stop the bullying (“external solution”), including “I would ask others for help” and “I would only be able to stop the bullying with the help of others”. The items per factor correlated moderately strongly (*r* = .55 for belief in internal solution, *r* = .49 for helplessness, and *r* = .41 for belief in external solution). We averaged the items for each of the three scales to assess students' cognitions about the solution to their situation. Higher scores on the three scales were indicative of higher *helplessness* and a stronger belief in an *internal solution* or an *external solution*, respectively.

#### Covariates: gender, age, victimization frequency at T1, and depressive symptoms at T1

At each wave, students were asked which gender they identified with and they could answer with ‘boy’, ‘girl’, or ‘other’. Information provided regarding students' gender at T1 and T2 was condensed into one dummy variable with girls (54.8%) being the reference category. A low percentage of students identified as neither boy nor girl (0.8%), so their values were treated as missing. Students' age was calculated as the difference in years between their date of birth and the start of the SOLID study (e.g., September 26, 2022, for cohort 1 and September 25, 2023, for cohort 2). Victimization frequency at T1 was added as a covariate in analyses.

*Depressive symptoms* were assessed at T1 using a shortened version of the Major Depressive Disorder Scale (MDDS), part of the Revised Child Anxiety and Depression Scale (Chorpita et al., 2000). It included five items shown to have similar discriminant and convergent validity as the longer version (Radez et al., 2021). Students reported on a 4‐point scale (0 = *never*, 1 = *sometimes*, 2 = *often*, 3 = *always*) how often they had experienced certain feelings in the past month, including: “*I felt sad and empty”*. Higher values were indicative of higher depressive symptoms. Confirmatory factor analysis produced a good fit (CFI = .999, TLI = .996, RMSEA = .030, SRMR = .008) and reliability was high with *ω* = .85. The five items were averaged to create a scale for depressive symptoms at T1.

### Analytic strategy

To examine the concurrent links between bystander behaviors (i.e., joining in the bullying and defending the victim, respectively) and the four cognitions (i.e., self‐blame, belief in internal and external solution, helplessness), we conducted linear regression analyses. Next, to examine the longitudinal role of bystander behaviors in victims' within‐person changes in cognitions, and the moderating role of changes in victimization, we ran two latent change score models (LCSM) per cognitive outcome. First, we analyzed the main effects of bystander behaviors at T1 predicting within‐person changes in cognitions between T1 and T2, while controlling for covariates. Second, we included the interaction terms between changes in victimization (i.e., the dichotomized difference score, representing a decrease vs. stability/increase in victimization frequency between T1 and T2) and bystander behaviors to assess the moderating role of changes in victimization in the link between bystander behaviors and within‐person changes in cognitions.

Both cross‐sectional and longitudinal analyses were conducted in lavaan 0.6.17 (Rosseel, 2012), in R version 4.3.1. Models are saturated with no degrees of freedom; therefore, no fit indices can be obtained. For all analyses, we applied the robust maximum likelihood estimation, estimated cluster‐robust standard errors (controlling for nestedness of victims within classrooms), and applied full information maximum likelihood (FIML) to handle missing data. A total of 34.8% of the victims had missing data on at least one of the variables of interest. Students with incomplete data were more frequently victimized at T1 (*p =* .005, Cohen's *d* = .23). There were no significant differences between complete and incomplete cases in any other variable used as predictor or outcome in the current study. We chose to not apply any method for controlling the family‐wise error rate as this study is the first to test the role of bystander behaviors in victims' cognitions. Given the exploration of these new relations, we prioritized achieving higher statistical power over strictly minimizing the risk of Type I error.

### Transparency and openness statement

The current study was pre‐registered (https://osf.io/hv5s2). Deviations from the (initial) pre‐registration are listed in Appendix A. The analytic code to reproduce the current study findings is available on https://github.com/lydialaningawijnen/JRAwhatsonyourmind. Pseudonymized data used for this study is available from the first author upon request, and the full dataset will be shared after the project has ended. We refrained from using AI in writing this manuscript.

## RESULTS

### Descriptive findings

Means, standard deviations, and correlations are presented in Table 1. Having bystanders join in the bullying was significantly related to higher self‐blame and helplessness, but correlations were weak in size. Having bystanders join in the bullying was unrelated to a belief in an internal or external solution. Having defenders was weakly negatively related to self‐blame at T1, and weakly positively related to a belief in an internal and external solution to the bullying. Moreover, having defenders was weakly negatively related to students' helplessness at T1. Students' self‐blame for victimization was weakly to moderately positively correlated with helplessness. Victim's belief in an external solution correlated positively with their belief in an internal solution. Helplessness was positively associated with the belief in an external solution and negatively correlated to a belief in an internal solution. Depressive symptoms at T1 were positively related to self‐blame and helplessness at T1 *and* T2, and negatively related to a belief in an internal and external solution at both T1 and T2 (all weak to moderate correlations).

**TABLE 1:** Means, standard deviations, and correlations between variables of interest in sample of victims (*n* ≈ 755).

Variables	*M*	*SD*	1.	2.	3.	4.	5.	6.	7.	8.	9.	10.	11.	12.	13.	14.
1. Bystanders join bully T1	0.49	0.50														
2. Bystanders defend victim T1	0.62	0.49	.03													
3. Victimization frequency T1	1.20	0.73	.**21**	**−.07**												
4. Victimization frequency T2	0.80	0.82	.**17**	.03	.**46**											
5. Self‐blame T1	1.24	0.79	.**13**	**−.08**	.**24**	.**16**										
6. Self‐blame T2	1.08	0.79	.**18**	.02	.**13**	.**25**	.**50**									
7. Internal solution T1	1.68	0.79	.01	.**19**	**−.09**	**−.11**	−.03	−.03								
8. Internal solution T2	1.68	0.77	.01	.08	−.07	−.04	**−.13**	−.05	.**47**							
9. External solution T1	1.41	0.78	.05	.**16**	.02	−.01	.**12**	.02	.**28**	.**12**						
10. External solution T2	1.36	0.79	.02	.**11**	.04	.06	.06	.**12**	.07	.**18**	.**42**					
11. Helplessness T1	1.34	0.81	.**15**	**−.12**	.**17**	.**19**	.**40**	.**27**	**−.20**	**−.26**	.**22**	.01				
12. Helplessness T2	1.23	0.81	.**13**	−.04	.**14**	.**35**	.**19**	.**40**	**−.22**	**−.20**	.**12**	.**31**	.**41**			
13. Boy	0.43	0.50	**−.05**	**−.13**	.06	−.02	−.04	−.06	.**09**	.**18**	−.03	−.02	**−.22**	**−.19**		
14. Age	12.75	1.77	.04	**−.27**	.**09**	.07	−.03	−.02	−.05	.02	**−.12**	**−.16**	−.06	−.06	.01	
15. Depressive symptoms T1	1.33	0.69	.**10**	−.06	.**26**	.**17**	.**32**	.**20**	**−.19**	**−.21**	**−.12**	**−.17**	.**39**	.**24**	**−.25**	.**15**

*Note*: Bolded estimates are significant with *p* < .05.

Interestingly, having defenders was unrelated to between‐person variation in victimization at T2 (*r* = .03). We also conducted a chi‐squared cross‐tabulation test to examine whether defended victims differed from non‐defended victims in the likelihood that they decreased in victimization (with at least −.20 points), or instead, remained stable or increased. Among non‐defended victims, 35.1% remained stable or increased in victimization, while 64.9% decreased in victimization. Among defended victims, 41.6% remained stable or increased in victimization, while 58.4% decreased in victimization. Defended victims and non‐defended victims did not significantly differ from each other in the likelihood that they decreased vs. remained stable/increased in victimization over time (*χ* ^2^(1) = 2.13, *p* = .145).

### The concurrent associations between bystander behaviors and victims' cognitions

Concurrent linear regression analyses reported in Table 2 indicate that victims whose bystanders *joined in the bullying* were more likely to blame themselves for the situation (St. Est. = 0.19) and experienced greater helplessness (St. Est. = 0.21) as compared with victims whose bystanders did not join in the bullying, consistent with our hypothesis. Victims with and without bystanders joining the bullying did not differ from each other in their belief in an internal solution (St. Est. = 0.06; which is not in line with our hypothesis) *nor* in their belief in an external solution (St. Est. = 0.07).

**TABLE 2:** Concurrent regression analysis for victims at T1 (*n* = 755).

	Self‐blame		Internal solution	
	Est (*SE*)	Std. Est	Est (*SE*)	Std. Est
Boy	0.02 (0.06)	0.02	**0.10 (0.06)**	**0.12**
Age	**−0.05 (0.02)**	**−0.12**	0.01 (0.02)	0.03
Bystanders join bully	**0.15 (0.05)**	**0.19**	0.05 (0.06)	0.06
Bystanders defend victim	**−0.13 (0.06)**	**−0.16**	**0.30 (0.06)**	**0.38**
Victimization frequency	**0.17 (0.04)**	**0.16**	−0.04 (0.06)	−0.04
Depressive symptoms	**0.33 (0.05)**	**0.28**	**−0.17 (0.05)**	**−0.16**

	External solution		Helplessness	
	Est (*SE*)	Std. Est	Est (*SE*)	Std. Est
Boy	−0.10 (0.06)	−0.10	**−0.25 (0.06)**	**−0.31**
Age	**−0.04 (0.02)**	**−0.08**	**−0.08 (0.02)**	**−0.17**
Bystanders join bully	0.07 (0.06)	0.07	**0.17 (0.05)**	**0.21**
Bystanders defend victim	**0.19 (0.06)**	**0.18**	**−0.27 (0.05)**	**−0.33**
Victimization frequency	0.08 (0.05)	0.08	**0.09 (0.04)**	**0.08**
Depressive symptoms	**−0.16 (0.05)**	**−0.14**	**0.40 (0.05)**	**0.34**

*Note*: Bolded estimates are significant with *p <* .05. *R* ^2^ are 15.0% for self‐blame, 7.0% for a belief in an internal solution, 5.2% for a belief in an external solution, and 22.7% for helplessness.

Regarding the concurrent role of *having bystanders defending the victim*, regression analyses indicated that defended victims experienced lower self‐blame (St. Est. = −0.16) and helplessness (St. Est. = −0.33) than non‐defended victims, *and* a stronger belief in an external solution (St. Est. = 0.18), which aligned with our hypotheses. Defended victims also more strongly believed in an internal solution than non‐defended victims (St. Est. = 0.38).

Regarding covariates, victimized boys experienced lower helplessness (St. Est. = −0.31) and reported a stronger belief that they could solve the bullying situation themselves (St. Est. = 0.12), as compared with victimized girls. With increasing age, victimized youth experienced lower self‐blame (St. Est. = −0.12), a weaker belief in an external solution (St. Est. = −0.08), and lower helplessness (St. Est. = −0.17). Victims with greater depressive symptoms reported higher self‐blame (St. Est. = −0.28) and helplessness (St. Est. = −0.34), and a weaker belief in an internal (St. Est. = −0.16) and external solution (St. Est. = −0.14). The concurrent models explained small to moderate proportions of the variance in the four cognitions (15.0% for self‐blame, 7.0% for internal solution, 5.2% for external solution, and 22.7% for helplessness).

### The role of bystander behaviors in victims' within‐person changes in cognitions

To investigate the role of bystander behaviors in victims' within‐person changes in cognitions, we ran four Change Score Models (for each cognition separately). These are all reported in Table 3.

**TABLE 3:** Latent change score models testing the role of bystander behaviors in within‐person changes in four types of cognitions in victims (*n* = 755).

	Self‐blame				Internal solution			
	Main effects		Interaction effects		Main effects		Interaction effects	
	Est. (*SE*)	St. Est.	Est. (*SE*)	St. Est.	Est. (*SE*)	St. Est.	Est. (*SE*)	St. Est.
Cognition T1 → Δ Cognition	**−0.53 (0.04)**	**−0.53**	**−0.53 (0.04)**	**−0.53**	**−0.58 (0.04)**	**−0.57**	**−0.57 (0.04)**	**−0.57**
Boy → Δ Cognition	−0.03 (0.06)	−0.04	−0.02 (0.06)	−0.03	**0.18 (0.06)**	**0.18**	**0.17 (0.06)**	**0.24**
Age → Δ Cognition	−0.001 (0.02)	−0.002	−0.000 (0.02)	−0.00	0.03 (0.02)	0.07	0.03 (0.02)	0.06
Victimization T1 → Δ Cognition	0.02 (0.06)	0.02	0.02 (0.06)	0.02	−0.04 (0.06)	−0.03	−0.04 (0.06)	−0.03
Depressive symptoms T1 → Δ Cognition	0.05 (0.05)	0.04	0.05 (0.05)	0.04	**−0.11 (0.05)**	**−0.10**	**−0.11 (0.05)**	**−0.10**
Bystanders join bullying → Δ Cognition	**0.17 (0.06)**	**0.25**	0.11 (0.11)	0.17	0.02 (0.06)	0.02	0.09 (0.10)	0.13
Bystanders defend victim → Δ Cognition	0.10 (0.06)	0.14	0.14 (0.11)	0.20	0.06 (0.06)	0.09	0.07 (0.10)	0.09
Decrease in victimization between T1 and T2	**−0.20 (0.06)**	**−0.20**	−0.21 (0.13)	**−0.30**	−0.02 (0.07)	−0.03	0.04 (0.12)	0.05
Bystanders join bullying → Decrease in victimization between T1 and T2 → Δ Cognition			0.09 (0.13)	0.05			−0.13 (0.12)	−0.07
Bystanders defend victim → Decrease in victimization between T1 and T2 → Δ Cognition			−0.06 (0.12)	−0.04			−0.01 (0.12)	−0.01
*R* ^2^ in Δ Cognition	28.6%		28.7%		31.7%		31.8%	

	External solution				Helplessness			
	Main effects		Interaction effects		Main effects		Interaction effects	
	Est. (*SE*)	St. Est.	Est. (*SE*)	St. Est.	Est. (*SE*)	St. Est.	Est. (*SE*)	St. Est.
Cognition T1 → Δ Cognition	**−0.60 (0.04)**	**−0.56**	**−0.60 (0.04)**	**−0.56**	**−0.67 (0.05)**	**−0.62**	**−0.67 (0.05)**	**−0.63**
Boy → Δ Cognition	−0.09 (0.07)	−0.09	−0.08 (0.07)	−0.11	**−0.14 (0.07)**	**−0.20**	−0.13 (0.07)	−0.18
Age → Δ Cognition	**−0.04 (0.02)**	**−0.09**	−0.04 (0.02)	−0.09	−0.03 (0.02)	−0.07	−0.03 (0.02)	−0.07
Victimization T1 → Δ Cognition	0.09 (0.06)	0.08	0.10 (0.07)	0.08	0.10 (0.07)	0.08	0.11 (0.07)	0.09
Depressive symptoms T1 → Δ Cognition	**−0.18 (0.05)**	**−0.15**	**−0.19 (0.05)**	−0.15	0.09 (0.06)	0.07	0.08 (0.06)	0.06
Bystanders join bullying → Δ Cognition	0.01 (0.07)	0.01	−0.04 (0.11)	−0.05	0.10 (0.06)	−0.07	−0.03 (0.10)	−0.04
Bystanders defend victim → Δ Cognition	0.03 (0.06)	0.04	**0.23 (0.11)**	0.31	−0.05 (0.07)	0.14	0.11 (0.10)	0.16
Decrease in victimization between T1 and T2	−0.04 (0.07)	−0.06	0.11 (0.14)	0.14	**−0.29 (0.07)**	**−0.41**	−0.24 (0.12)	−0.13
Bystanders join bullying × Decrease in victimization between T1 and T2 → Δ Cognition			0.09 (0.13)	0.05			0.22 (0.12)	0.11
Bystanders defend victim × Decrease in victimization between T1 and T2 → Δ Cognition			**−0.33 (0.14)**	**−0.18**			**−0.27 (0.13)**	**−0.15**
*R* ^2^ in Δ Cognition	30.4%		31.2%		34.2%		35.2%	

*Note*: Δ = Latent change between T1 and T2. Bolded estimates are significant with *p* < .05.

#### Self‐blame

On average, T1 victims decreased in self‐blame over time, with *unconditional M* Δ = −0.16 (*SE* = 0.03), *p* < .001. In line with our hypothesis, victims whose bystanders joined in the bullying experienced *less* favorable changes in self‐blame over time (i.e., stronger increase, or weaker decrease) than victims whose bystanders did not join in the bullying (Est = 0.17, *SE* = 0.06, *p* = .007). In contrast to our hypothesis, models including interaction terms revealed that this effect was *not* exacerbated if students decreased in victimization (Est = 0.09, *SE* = 0.13, *p* = .495). Defended victims did not significantly differ from non‐defended victims in within‐person changes in self‐blame (Est = 0.10, *SE* = 0.06, *p* = .096). The longitudinal role of being defended in changes in self‐blame was *not* moderated by the extent to which victimization decreased over time (vs. remained stable/decreased), which is inconsistent with our hypothesis (Est = −0.06, *SE* = 0.12, *p* = .630). Yet, there was a main effect of changes in victimization: victims who decreased in victimization were more likely to decrease in self‐blame over time as compared with victims who remained stable or increased in victimization (Est = −0.20, *SE* = 0.06, *p* = .001).

Regarding covariates, victims who reported higher self‐blame at T1 experienced more favorable changes (i.e., stronger decrease, or less strong increase) in self‐blame over time (Est = −0.53, *SE* = 0.04, *p* < .001), but there were no significant effects of age, gender, victimization frequency, or depressive symptoms on within‐person changes in self‐blame (Table 3).

#### Belief in internal solution

On average, victims did not significantly change over time in their belief that—if they would be bullied—they could solve the situation themselves (internal solution), with *unconditional M* Δ = −0.01 (*SE* = 0.03), *p* = .709. Having bystanders join the bullying did *not* significantly predict within‐person changes in the belief in an internal solution over time (Est = 0.02, *SE* = 0.06, *p* = .819), and the moderation effect of changes in victimization was not significant either (Est = −0.13, *SE* = 0.12, *p* = .305). Thus, our hypothesis that victims whose bystanders joined in the bullying would decrease more in their belief in an internal solution than victims whose bystanders do *not* join the bullying was rejected in favor of the null hypothesis. We also explored the longitudinal role of having defenders in within‐person changes in an internal solution, which was non‐significant, as was the moderating effect of changes in victimization.

Regarding covariates, victimized boys experienced more favorable changes (stronger increase, weaker decrease) in their belief in an internal solution than victimized girls (Est = 0.18, *SE* = .06, *p* = .005). Greater depressive symptoms at T1 were linked to less favorable changes in a belief in an internal solution (Est = −0.11, *SE* = 0.05, *p* = .031). Age and victimization frequency at T1 were unrelated to changes in a belief in an internal solution (Table 3).

#### Belief in external solution

On average, victims did not significantly change in their belief that there would be an external solution to their situation over time, with *unconditional M* Δ = −0.06 (*SE* = 0.04), *p* = .077. The main effect models revealed that having bystanders join the bullying (Est = 0.01, *SE* = 0.07, *p* = .915), or having defenders (Est = 0.03, *SE* = 0.03, *p* = .651), or changes in victimization (Est = −0.04, *SE* = 0.07, *p* = .575) had no significant effect on changes in a belief in an external solution. Regarding covariates, with increasing age, students decreased (or were less likely to increase) in a belief in an external solution. Higher depressive symptoms were linked to a decrease (or less strong increase) in a belief in an external solution (Est = −0.18, *SE* = 0.05, *p* = .001). There were no significant effects of gender and victimization frequency at T1 (Table 3).

**FIGURE 2:**
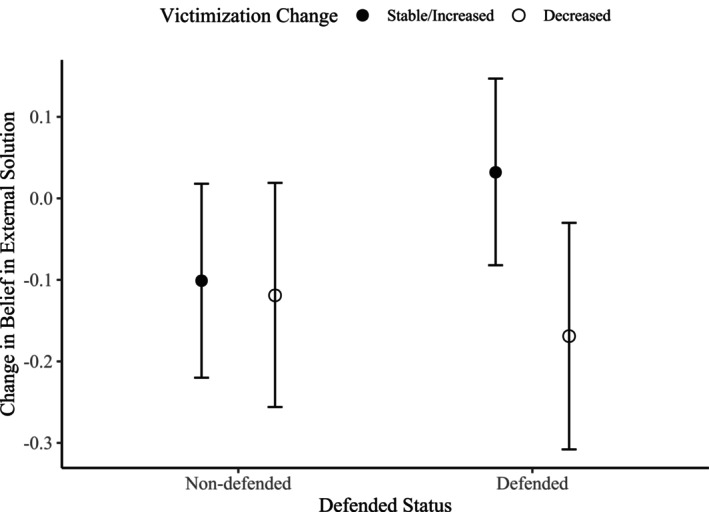
Standardized estimated parameters and confidence intervals of *change* in a belief in an external solution for non‐defended vs. defended victims who remain stable/increase vs. decrease in victimization. Only defended victims who decreased in victimization significantly *decreased* in a belief in an external solution (St. Est = −0.17, *SE* = 0.07, *p* = .017).

In interaction models, there was a significant interaction between having defenders and changes in victimization (Est = −0.33, *SE* = 0.14, *p* = .020). The main effect of being defended reached significance in the model including interactions, suggesting that defended victims who remain stable or increase in victimization more strongly *increase* (or less strongly decrease) in a belief in an external solution to the bullying (Est = 0.23, *SE* = 0.11, *p* = .041) than non‐defended victims who remain stable or increase in victimization. In Figure 2, we display standardized estimated parameters and 95% confidence intervals, which indicate that defended victims who decrease in victimization significantly *decrease* in a belief in an external solution (St. Est = −0.17, *SE* = 0.07, *p* = .017), while the other three groups—that is, (non‐)defended victims remaining stable/increasing in victimization and non‐defended victims decreasing in victimization—did not significantly change over time in their belief in an external solution (with *p*'s varying from .091 to .579), which contrasts our hypothesis. The model including interactions predicted only 0.8% more variance than the model that only included main effects, suggesting a very small effect that should be interpreted with caution.

#### Helplessness

**FIGURE 3:**
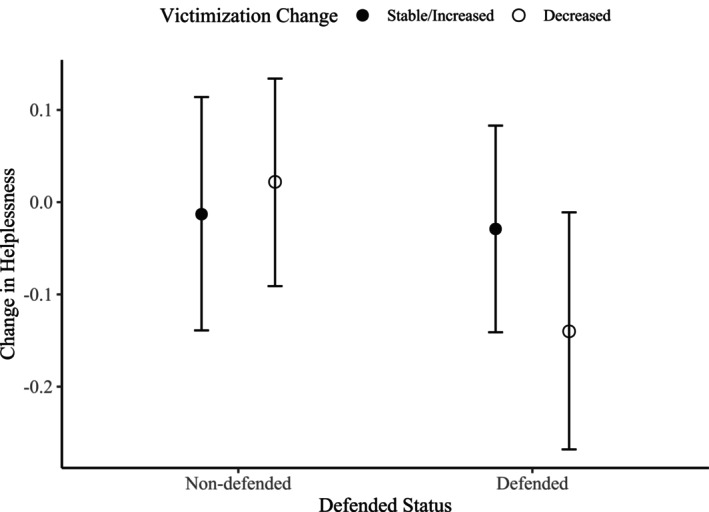
Standardized estimated parameters and confidence intervals of *change* in helplessness for non‐defended vs. defended victims who remain stable/increase vs. decrease in victimization. Only defended victims who decreased in victimization significantly decreased in helplessness (St. Est. = −0.14, *SE* = 0.07, *p* = .033).

On average, victims significantly decreased in helplessness over time, with *unconditional M* Δ = −0.11 (*SE* = 0.04), *p* < .003. Victims who decreased in victimization experienced more favorable changes in helplessness than victims who remained stable or increased in victimization (Est = −0.29, *SE* = 0.07, *p* < .001). Victims whose bystanders joined the bullying did not significantly differ from victims whose bystanders did *not* join the bullying in within‐person changes in helplessness, irrespective of changes in victimization between T1 and T2. This finding was in contrast to our hypotheses that victims whose bystanders joined the bullying would report increased helplessness, especially if their victimization increased. Further, there was *no* main effect of being defended on within‐person changes in helplessness (Est = −0.05, *SE* = 0.07, *p* = .446). Yet, there was a significant interaction effect between being defended and decreasing victimization (Est = −0.27, *SE* = 0.13, *p* = .039). Estimated standardized parameters and confidence intervals are displayed in Figure 3. Only defended victims who decreased in victimization significantly decreased in helplessness (St. Est. = −0.14, *SE* = 0.07, *p* = .033), which aligns with our hypothesis; while other groups did not significantly change in helplessness (with *p*'s varying from .613 to .847). The model including interactions only explained 1.0% more variance than the model including only main effects, suggesting a very small effect that should be interpreted with caution. Regarding covariates, victimized boys declined more strongly in helplessness than victimized girls, while the other covariates did not significantly predict changes in helplessness.

### Sensitivity analyses

In our analyses described above, we applied a researcher‐defined cut‐off to distinguish victims who (1) decreased versus (2) were stable/increased in victimization, which enabled testing our hypotheses. However, categorizing variables comes with the cost of loss of information (e.g., about the degree of change). Therefore, as a sensitivity analysis, we examined whether the findings of LCSM remained the same when replacing the dichotomized change score with the original continuous change score for victimization (which ranged from −3.10 to 4.00, with negative scores reflecting an increase, zero representing stability, and positive scores reflecting decreases in victimization). All models using the continuous change score are reported in Appendix S2 (Table S1). In general, results with the continuous change score were highly similar to the results obtained with the categorical change score, with two exceptions. One additional significant interaction emerged for helplessness, suggesting that students who decreased in victimization were more likely to decrease in helplessness between T1 and T2, unless they had bystanders join in the bullying at T1 (Est = 0.18, *SE* = 0.07, *p* = .014). Estimated marginal effects suggest that students who did not have bystanders joining the bullying and who decreased in victimization were most likely to decrease in helplessness (Est = −0.15, *SE* = 0.05, *p* = .001), while other groups remained stable (*p*'s varying from .244 to .408). Moreover, an additional effect emerged for *victimization frequency*: students who were more frequently victimized at T1 were more likely to increase (or less likely to decrease) in helplessness between T1 and T2.

## DISCUSSION

It is widely assumed that victims' maladaptive cognitions regarding the cause of their victimization or its solution play a crucial role in the development of psychological problems (Perren et al., 2013). However, research investigating the conditions under which victims develop such maladaptive cognitions remains limited. This study examined whether bystanders' responses in bullying situations influence victims' cognitions about the cause (self‐blame) and the solution (internal, external, or no solution) to bullying, both concurrently and longitudinally. Regarding cognitions about the *cause* (self‐blame), our concurrent findings suggest that victims whose bystanders join the bullying have higher self‐blame than victims whose bystanders do *not* join the bullying. Longitudinal analyses demonstrated that these differences persisted over time, with victims whose bystanders joined the bullying being less likely to decrease in self‐blame than victims whose bystanders did *not* join the bullying. Moreover, victims who were defended experienced *lower* self‐blame than non‐defended victims, concurrently. Yet, longitudinally, defended victims did not differ from non‐defended victims in the extent to which they decreased in self‐blame over time.

Regarding cognitions about the *solution*, concurrent findings revealed that victims whose bystanders joined the bullying reported greater helplessness, perceiving no viable solution to their victimization. Conversely, defended victims were more likely to believe in a solution, either through personal coping strategies (internal solution) or external support (external solution), and reported lower helplessness. Longitudinal findings suggested that defended victims decreased in helplessness *and* in a belief in an external solution over time, but only if their victimization also decreased between T1 and T2—although effects were very small in size.

### Do bystander behaviors predict victims' cognitions about the *cause* of the bullying?

Our findings suggest that bystanders' behaviors shape victims' attributions regarding the cause of their victimization. Victims whose bystanders *joined the bullying* were more likely to blame themselves for the situation concurrently, and they were less likely to decrease (or even more likely to increase) in self‐blame over time. This observation aligns with attribution theory (Weiner & Graham, 1999), which suggests that when others attribute a problem to a lack of effort of the person experiencing it (internal, controllable), this leads to reprimands (Weiner, 2000). When victims notice that bystanders join in the bullying, they may perceive this as a ‘reprimand’, signaling peers' convictions that the victims are to blame for the situation. Additionally, the presence of bystanders during bullying episodes may worsen victims' feelings of humiliation due to the ‘spectator effect’, which potentially increases self‐blame over time (Menesini & Camodeca, 2008). Moreover, upward social comparisons (Visconti et al., 2013) may have fostered self‐blame in victims whose bystanders joined the bullying.

Moreover, victims whose bystanders *defended* them experienced concurrently lower self‐blame than victims whose bystanders did *not* defend them—perhaps because bystander intervention signals that the bullying behavior is perceived as unjust and that the responsibility lies with the aggressor rather than the victim (Laninga‐Wijnen et al., 2024b). Yet, the concurrent link between being defended and self‐blame could *also* indicate that victims who are less likely to blame themselves for the victimization were more willing to ask for help from peers and thus, were more likely to be defended (Visconti et al., 2013). Longitudinally, victims experienced within‐person reductions in self‐blame, irrespective of whether they were being defended or not at T1. Furthermore, in contrast to our hypothesis, the role of being defended in within‐person changes in self‐blame over time was *not* moderated by within‐person changes in victimization. We had anticipated a *decrease* in self‐blame for defended victims who declined in victimization, and an *increase* in self‐blame for defended victims who remained stable or increased in victimization. It is possible that the effect of being defended on self‐blame occurs very quickly (given the concurrent finding) and that after this, defended victims follow a similar development in self‐blame as non‐defended victims—irrespective of the course of their victimization. Indeed, a daily diary study showed that victims reported lower self‐blame on days that they are being defended as compared with on days that they are not being defended, suggesting that bystander behaviors may promptly affect victims' cognitions (Laninga‐Wijnen, Garandeau, et al., 2024). This daily diary study was cross‐sectional, however; thus future work is encouraged to examine such daily processes longitudinally, and evaluate whether the *consistency* of being defended across victimization events may mitigate self‐blaming tendencies over time.

### Do bystander behaviors predict victims' cognitions about the *solution* to the bullying?

Regarding *belief in an internal solution*, concurrent findings revealed that being defended was related to greater confidence in victims that, if bullied, they would be able to handle the situation themselves, which is consistent with our hypothesis. However, there was no effect on belief in an internal solution from having bystanders join the bullying, which contrasts with our hypothesis. There were no longitudinal effects of bystander behaviors and/or changes in victimization in beliefs about an internal solution. The observed concurrent associations between being defended and a belief in an internal solution align with self‐efficacy theory (Bandura, 1997), suggesting that defended victims may feel empowered and learn vicariously from their defenders, and thus, believe that they will also be able to stand up for themselves. Yet, given that we did not detect a longitudinal effect of defending on changes in an internal solution, it is likely that either the effect of defending occurs very *quickly* (e.g., across days, rather than across months), or the effect operates in the *reverse direction*. That is, victims who believe that they can stand up for themselves (internal solution) when being bullied may also be more likely to do that, and therefore more clearly show to bullies that they do not like the bullying behaviors they are subjected to, which potentially activates bystander defending (Laninga‐Wijnen et al., in press).

With respect to *belief in an external solution*, being defended was concurrently related to a stronger belief that the situation could be solved with the help of others, which corresponds to our hypothesis. However, longitudinal findings revealed that defended victims whose victimization *decreased* over time significantly declined in their belief in an external solution, while other groups remained relatively stable in this belief. This pattern contrasts with our initial hypothesis, which anticipated an increase in the belief in an external solution among defended students whose victimization declined (because we anticipated that in these cases, victims may have perceived the defending behavior as effective). Although the effect size was small and should be interpreted with caution, several possible explanations may account for this unexpected finding.

One possibility is that defended students who experienced reduced victimization may have internalized coping strategies modeled by their defenders, leading them to feel more self‐reliant and less dependent on external help in the future. However, given the absence of a longitudinal effect of being defended on changes in their belief in an internal solution, this interpretation appears less likely. An alternative explanation is that defended victims who decrease in victimization did not attribute the decrease in victimization to the defending behavior itself (and thus, to the external help of others), but rather to other factors—some of which may have been indirectly prompted by the defending. For example, the defending might have triggered insecurity in bullies, which may lead defended victims to believe that bullies ceased their behavior due to increased insecurity (rather than that bullies stopped in response to the defending of peers). Another possibility is that defended students whose victimization decreases regain self‐esteem and agency, and may come to view seeking help as unnecessary or even stigmatizing, thereby reducing their belief in the usefulness of external solutions. Alternatively, it may be that defender behavior tended to occur following attempts of the victimized student to stand up for themselves (Laninga‐Wijnen et al., in press), so the victimized student attributed success to their own efforts. Lastly, it is possible that the two‐item scales offered insufficient information to clearly distinguish between victims' belief in an internal and external solution. In our data, there is a small positive correlation between the two, suggesting that both beliefs may co‐exist in individuals. Future work is encouraged to develop more comprehensive questionnaires to better disentangle students' belief in an internal and/or external solution to their situation. Moreover, future research is encouraged to investigate the role of being defended on short‐term changes in students' belief in internal versus external solutions. Employing high‐frequency, time‐sensitive designs (e.g., daily diary or experience sampling methods) could provide better insights into the temporal dynamics of these beliefs.

Regarding *helplessness*, in line with our hypothesis, concurrent findings revealed that victims whose bystanders joined the bullying experienced greater helplessness (i.e., thought that if they would be bullied, there would be no solution to that) than victims whose bystanders did not join the bullying. When bystanders join the bullying, the situation may be experienced as more threatening (primary appraisal) and thus, harder to solve, which aligns with the Transactional Model of Stress and Coping (Lazarus & Folkman, 1984). In addition, victims whose bystanders defended them concurrently experienced lower helplessness than non‐defended victims, possibly because receiving support may diminish the extent to which a situation is perceived as threatening or challenging (primary appraisals) and may increase hope that the situation will be solved by highlighting that there are coping resources available (Gazelle & Druhen, 2009; Ricker et al., 2022).

Longitudinal findings revealed that defended victims only decreased in helplessness if they also decreased in victimization over time, which aligns with our hypothesis, although the effect was small in size. Thus, in the longer term, the effectiveness of being defended in decreasing helplessness depended on whether the initial hope that was triggered by defending behaviors was fulfilled (i.e., hope that victimization would decrease). Notably, our descriptive findings suggest that defended victims were *not* more likely to decrease in victimization than non‐defended victims: 64.9% of the non‐defended victims decreased in victimization, while only 58.4% of the defended victims decreased in victimization, and the differences in these percentages were not significant. This finding aligns with previous work detecting no relation between being defended and changes in victimization (Laninga‐Wijnen et al., 2023). This however does *not* imply that being defended is ineffective for all students. It is possible that under certain conditions (e.g., skills or characteristics of bystander, timing of intervention, relationship between defender and victim), being defended reduces victimization, which in turn diminishes victims' helplessness. Future work is encouraged to examine what factors optimize the effectiveness of being defended in reducing victimization, because this reduction in victimization seems to contribute to decreasing helplessness in victims (although the effect may be small). Moreover, future work could examine why non‐defended victims decrease in helplessness irrespective of whether their victimization decreases. This may offer insights into other potential resources of resilience for non‐defended victims.

### Covariates: Gender, age, victimization frequency, and depressive symptoms

In comparison to victimized girls, victimized boys experienced stronger decreases in helplessness and increases in a belief in an internal solution, suggesting that boys are more confident that their situation will change and experience greater agency related to this solution. Victimized boys and girls did not significantly differ in (changes in) self‐blame, which contrasts with a recent study that found boys to be less likely to experience self‐blame in socially ambiguous situations (Di Tata et al., 2025), but aligns with another study showing that peer victimization related to greater self‐blame among girls than among boys (Singh & Bussey, 2011). Regarding age, older victimized students were less likely to report an increased belief in an external solution and more likely to report lower helplessness. In line with previous work, more frequently victimized youth blamed themselves more for victimization (Laninga‐Wijnen, Garandeau, et al., 2024) and experienced greater helplessness (cf. Ricker et al., 2022), although we did not detect longitudinal effects of victimization frequency at T1 after including changes in victimization between T1 and T2 as predictor (except in sensitivity analyses, in which more frequently victimized youth tended to increase in helplessness over time). Lastly, depressive symptoms were concurrently positively linked with self‐blame (cf. Schacter & Juvonen, 2017) and helplessness (cf. Nixon et al., 2023), and both concurrently and longitudinally related to a decreased belief in internal and external solutions.

### Strengths and limitations

The current study has several strengths. It tested a question of high theoretical significance for our understanding of bullying, bystander behaviors, and their consequences, using both a cross‐sectional and longitudinal design and a large sample of victims. There were also several limitations. Some pertain to our questionnaire assessing victims' belief in a solution. Although exploratory factor analyses suggested that a three‐factor solution was preferred over a one‐ and two‐factor solution, the three scales of this self‐developed measure only included two items each, which warrants caution in interpreting our research findings. More items per factor may need to be added in future work to better distinguish between students' belief in an internal, external, or *no* solution. Moreover, although the main purpose of the scale was to assess *cognitions* (about what students think would be the solution if they would be bullied), it is unknown whether all items truly assessed such *cognitions* (beliefs) or instead, *coping*, that is, the things that students *actually did when they had been bullied*. Although students' cognitions and actual coping behaviors are probably strongly intertwined, further research is needed to better disentangle students' cognitions from their actual behaviors in response to bullying situations.

Another important avenue for future research is to identify what cognitions are maladaptive, and when, by examining the psychological consequences of such cognitions (cf. Singh & Bussey, 2011). The current study provides a first step in understanding the (mal)adaptive nature of these cognitions by revealing that depressive symptoms are concurrently positively linked with self‐blame and helplessness, and both concurrently and longitudinally related to a decreased belief in internal and external solutions. Future work is needed to disentangle under what circumstances these cognitions may be maladaptive *or* adaptive and contribute to favorable psychological development.

Furthermore, the pre‐registered cut‐off for categorizing within‐person changes in victimization (decrease vs. stable/increase) could, as any researcher‐defined cut‐off, be considered somewhat arbitrary. We decided to use a cut‐off so that we could properly test our hypotheses about the distinction between victims who decreased, versus remained stable, or increased, in victimization. By using this criterion, we could capture all potential decreases: a decrease of .20 meant that the student experienced a 1‐point decrease in at least one out of the five victimization items. However, we cannot be certain that a decrease of .20 was actually distinguished or felt by students, and thus, whether this distinction can be considered meaningful. Yet, in our sensitivity analyses that evaluated the degree of change in victimization as a moderator, most effects remained the same.

Next, in our questionnaire, we did not have an option for students to indicate *that no bystanders were present*, although this might have been the case. It is likely that, in such cases, they selected ‘not applicable’, and were therefore excluded from our victim sample. However, it is also possible that victims without witnesses indicated that they were *not* defended, and thus were categorized as undefended victims. Previous work has shown that victims without witnesses are typically similar in adjustment to victims with defenders (Laninga‐Wijnen et al., 2024b). Yet, in the current study, they were included in the same group of non‐defended victims, which could have blurred potential differences between defended and non‐defended victims. In addition, our data did not allow us to identify the specific bystanders involved. Victims' reactions may depend on their relationship with the bystander—particularly if the bystander is someone they perceive as a friend or otherwise close to them.

Furthermore, this study was observational rather than experimental. We did not manipulate the independent variable of bystander actions. Therefore, the (lack of) relations between measured variables may have been impacted by other unmeasured variables. This limitation could be addressed in future research by implementing a matching procedure: defended victims could be matched to non‐defended victims based on potentially important variables (such as their psychological functioning, gender, age, and social adjustment), to allow reliable comparisons of whether bystander behaviors affect victims' cognitions. Moreover, future research should investigate how classroom‐ or school‐level factors may influence the impact of bystander behaviors on victims' cognitions. For instance, previous work has shown that victims experience more psychological problems in classrooms where victimization is less common (Laninga‐Wijnen et al., 2025)—a phenomenon coined the *healthy context paradox*. Researchers have suggested that this may be partially due to victims blaming themselves more for the victimization (e.g., “I am the only one being bullied because I am not a fun child”), although this presumption has not been rigorously tested. Future work is encouraged to examine how various cognitions may underlie the healthy context paradox—and how bystander behaviors may affect these dynamics.

A final limitation is that a higher participation rate would have been preferred, although our participation rate of 56% is comparable to other large‐scale longitudinal projects that applied active consent procedures (see Shaw et al., 2015, for a review). The main reason why our participation rate was not higher was that parents did not return consent forms, despite all our efforts and reminders to retrieve them. Although active consent is desirable for ethical reasons, it may come at the cost of obtaining a representative sample. One study found that victims were not under‐represented in studies applying active consent procedures as compared with passive consent procedures (Shaw et al., 2015), but future work is encouraged to better understand how active consent may limit reliable detection of effects.

### Conclusions and practical implications

Our findings indicate that bystanders' behaviors are concurrently related to victims' cognitions about the cause and the solution of their situation. Over time, victims whose bystanders joined the bullying were less likely to decrease in self‐blame than victims whose bystanders did not join in the bullying, and defended victims decreased more strongly in helplessness than non‐defended victims but only if their victimization also decreased. The concurrent findings suggest that *either* the effect of bystander behaviors on victims' cognitions occurs very quickly, *or* that victims' cognitions may predict how they behave during bullying situations, which in turn triggers certain bystander responses. Research examining the role of bystander behaviors in victims' cognitions over shorter time‐spans, for example with experience‐sampling designs, is needed to establish the temporal precedence of effects.

Anti‐bullying programs should emphasize—or continue to emphasize—how important it is for all students to not join the bullying in any way, as this contributes to increasing maladaptive cognitions (self‐blame) among victims. In addition, being defended does seem to have some beneficial effects on victims when their victimization also decreases—but more research is needed to examine under what conditions being defended does actually result in decreasing victimization. For instance, the effectiveness of defending in reducing victimization might vary depending on characteristics of the defender (e.g., their popularity), the bullying perpetrator (e.g., their motivations for targeting a victim), the victim themselves (e.g., whether they react aggressively to bullying) or the classroom (e.g., anti‐bullying norms). It is crucial to identify the factors that optimize the effectiveness of peers' defending behaviors, so that more victims will be able to develop adaptive cognitions and ultimately escape their plight.

## AUTHOR CONTRIBUTIONS

**Lydia Laninga‐Wijnen:** conceptualization, methodology, data curation, investigation, validation, formal analysis, funding acquisition, visualization, project administration, resources, writing – original draft. **Daniël Graf:** conceptualization, writing – original draft. **Karyn Healy:** conceptualization, writing – original draft. **Takuya Yanagida:** formal analysis, visualization, methodology. **Christina Salmivalli:** conceptualization, writing – original draft. **Claire F. Garandeau:** conceptualization, writing – original draft, validation.

## FUNDING INFORMATION

Lydia Laninga‐Wijnen was supported by a Postdoctoral Researcher Grant (No. 349560) from the Academy of Finland. There was no role of funding source in design, analysis, or writing of the report.

## CONFLICT OF INTEREST STATEMENT

The authors report no conflict of interest in the conduct and reporting of the research.

## ETHICS STATEMENT

The study was approved by the Ethical Board of the University of Turku in Finland (November 2021, reference number 53/2021).

## CONSENT STATEMENT

Active consent was obtained from parents, and assent from students.

## Supporting information


Appendix S1.

## Data Availability

Syntaxes will be retrieved from (LINK will be generated upon completion of analyses). Pseudonymized data will be made publicly available upon completion of the SOLID project (end 2025). Until then, the data that support the findings of this study are available from the corresponding author upon reasonable request.

## APPENDIX A (Deviations from Pre‐registration)

We pre‐registered our study before data collection was completed, but decided to adapt the pre‐registration after some reconsiderations in hypothesis formulation. Specifically, we deviated from our initial pre‐registration in two ways.

1. First, we initially had pre‐registered that we would examine the longitudinal role of bystander behaviors on within‐person changes in cognitions *only* in a sample of stable, frequently victimized youth. However, we realized that this hindered us in examining the role of bystander behaviors on within‐person changes in cognitions for the *full sample of victims—*including those whose defending decreased. Therefore, we adapted our pre‐registration by including *changes in victimization* (decrease vs. stability/increase) as a moderator. This way we could not only compare defended vs. non‐defended stable victims but also defended vs. non‐defended non‐stable victims, providing better insight to our hypotheses.
2. Second, we had pre‐registered that we would select students as victims if they had indicated that they were bullied once or twice on at least one of the five victimization items. Based on reviewer feedback of our previous papers on the same project, we decided to use a somewhat stricter criterion to identify youth as peer victims. We now selected students as victims if they had indicated that they were bullied once or twice on at least *two* out of the five victimization items, or if they indicated they had been bullied multiple times a month on at least one out of five items. We felt that being stricter in our criterion was especially important in this paper, because we were interested in changes in victimization as a moderator. We anticipated that if students would change from being bullied once or twice in one way to being a non‐victim, this may not be a very meaningful change—it is likely that they barely experienced bullying in the first place, and thus, any change may not feel like a change. Therefore, we decided to use a stricter criterion to define students as victims—so that any change in bullying is more likely to be ‘felt’.

Besides the changes in the pre‐registration, we deviated from the adapted pre‐registration in one way:

3 Because research on cognitions related to the solution of bullying is relatively new, we decided to include depressive symptoms as predictors, in order to better understand to which extent certain cognitions can be considered as (mal)adaptive. Models *without* depressive symptoms render similar results for all other predictors (can be retrieved upon request).
