# Supplementary material for: What's on your mind? The role of bystander behaviors in victims' cognitions about the cause of the bullying and its solution
Source: J Res Adolesc. 2026 Mar 30;36(2):e70172. doi: 10.1111/jora.70172 (PMC13034662; doi:10.1111/jora.70172)
Supplement: Supplementary file 1 — Appendix S1. [file JORA-36-0-s001.docx]

**Supplemental Materials**

**Supplemental Material 1 - Selection of Victims**

From the full sample of 6,357 students, we selected students as victim of bullying if they: 1) experienced one out of five types of victimization at two or more times a month, or 2) experienced two or more types of victimization at least once or twice in the past few months. This resulted in a sample of *n* = 961 victims. However, victims were excluded from this sample if they consistently reported on follow-up items, about bystander behaviors, that nobody had been mean to them nor bullied them (*n* = 206; cf. MASKED). We ended up with a sample of *n* = 755 victims. These victims were more frequently victimized (M = 1.20) than the 206 excluded victims (M = .84), Cohen’s *d* = .52 at T1. The 755 victims were also significantly younger (M = 13.36 vs. M = 12.75), Cohen’s *d* = -.36.

**Supplemental Material 2 - Sensitivity Analysis**

| Table S1  *Latent Change Score Models Testing the Role of Bystander Behaviors in Within-Person Changes in Four Types of Cognitions in Victims (n* = *755*) | | | | | | | | |
| --- | --- | --- | --- | --- | --- | --- | --- | --- |
|  | Self-blame | | | | Internal Solution | | | |
|  | Main effects | | Interaction effects | | Main effects | | Interaction effects | |
|  | *B(SE)* | *β* | *B(SE)* | *β* | *B(SE)* | *β* | *B(SE)* | *β* |
| Cognition T1 → Δ Cognition | **-0.53(0.04)** | **-0.53** | **-0.53(0.04)** | **-0.53** | **-0.57(0.04)** | **-0.57** | **-0.57(0.04)** | **-0.56** |
| Boy → Δ Cognition | -0.03(0.06) | -0.05 | -0.03(0.06) | -0.04 | **0.18(0.06)** | **0.27** | **0.17(0.06)** | **0.23** |
| Age → Δ Cognition | -0.004(0.02) | -0.01 | -0.004(0.02) | -0.01 | 0.03(0.02) | 0.06 | 0.03(0.02) | 0.06 |
| Victimization T1 → Δ Cognition | 0.09(0.07) | 0.08 | 0.09(0.07) | 0.08 | -0.02(0.07) | -0.10 | -0.03(0.07) | -0.02 |
| Depressive symptoms T1 → Δ Cognition | 0.05(0.05) | 0.04 | 0.05(0.05) | 0.04 | **-0.11(0.05)** | **-0.10** | **-0.11(0.05)** | **-0.10** |
| Bystanders join bullying → Δ Cognition | **0.15(0.06)** | **0.23** | **0.15(0.07)** | **0.21** | 0.01(0.06) | 0.02 | 0.05(0.07) | 0.07 |
| Bystanders defend victim → Δ Cognition | 0.10(0.06) | 0.15 | 0.12(0.07) | 0.17 | 0.06(0.06) | 0.09 | 0.08(.0.07) | 0.11 |
| Favorable change in victimization between T1-T2 | **-0.19(0.05)** | **-0.17** | -0.17(0.09) | -0.17 | -0.05(0.05) | -0.04 | 0.04(0.08) | 0.04 |
| Bystanders join bullying* Favorable change in victimization between T1-T2 → Δ Cognition | -- | -- | 0.01(0.09) | 0.01 |  |  | -0.11(0.09) | -0.08 |
| Bystanders defend victim* Favorable change in victimization between T1-T2 → Δ Cognition | -- | -- | -0.05(0.08) | -0.04 |  |  | -0.06(0.08) | -0.04 |
| *R*^2^ in Δ Cognition | 30.3% |  | 30.3% |  | 31.8% |  | 32.1% |  |
|  | External Solution | | | | Helplessness | | | |
|  | Main effects | | Interaction effects | | Main effects | | Interaction effects | |
|  | *B(SE)* | *β* | *B(SE)* | *β* | *B(SE)* | *β* | *B(SE)* | *β* |
| Cognition T1 → Δ Cognition | **-0.60(0.04)** | **-0.56** | **-0.59(0.04)** | **-0.55** | **-0.69(0.05)** | **-0.64** | **-0.70(0.05)** | **-0.65** |
| Boy → Δ Cognition | -0.09(0.07) | -0.13 | -0.09(0.07) | -0.11 | **-0.15(0.07)** | **-0.23** | **-0.15(0.07)** | **-0.18** |
| Age → Δ Cognition | **-0.05(0.02)** | **-0.09** | **-0.04(0.02)** | **-0.09** | **-0.04(0.02)** | **-0.08** | **-0.04(0.02)** | **-0.08** |
| Victimization T1 → Δ Cognition | 0.13(0.08) | 0.11 | 0.13(0.08) | 0.11 | **0.22(0.07)** | **0.18** | **0.23(0.07)** | **0.19** |
| Depressive symptoms T1 → Δ Cognition | **-0.18(0.05)** | **-0.15** | **-0.19(0.05)** | -0.15 | 0.09(0.05) | 0.07 | 0.09(0.05) | 0.07 |
| Bystanders join bullying → Δ Cognition | 0.001(0.07) | 0.002 | -0.02(0.08) | -0.03 | 0.07(0.06) | 0.10 | 0.02(0.07) | 0.02 |
| Bystanders defend victim → Δ Cognition | 0.03(0.06) | 0.04 | 0.12(0.07) | 0.15 | -0.05(0.07) | -0.08 | -0.002(0.07) | -0.003 |
| Favorable change in victimization between T1-T2 | -0.08(0.06) | -0.08 | -0.01(0.10) | -0.01 | **-0.31(0.05)** | -0.29 | **-0.33(0.07)** | **-0.30** |
| Bystanders join bullying* Favorable change in victimization between T1-T2 → Δ Cognition |  |  | 0.09(0.09) | 0.06 |  |  | **0.18(0.07)** | **0.12** |
| Bystanders defend victim* Favorable change in victimization between T1-T2 → Δ Cognition |  |  | **-0.24(0.05)** | **-0.17** |  |  | **-0.15(0.07)** | **-0.10** |
| *R*^2^  in Δ Cognition | 30.8% |  | 32.1% |  | 38.4% |  | 39.6% |  |
| *Note.*  Δ = Latent change between T1 and T2. Bolded estimates are significant with *p* < .05. Favorable change in victimization = stronger decrease or less strong increase in victimization between T1 and T2, and zero = stability. | | | | | | | | |
